# Serverless OpenHealth at data commons scale—traversing the 20 million patient records of New York’s SPARCS dataset in real-time

**DOI:** 10.7717/peerj.6230

**Published:** 2019-01-15

**Authors:** Jonas S. Almeida, Janos Hajagos, Joel Saltz, Mary Saltz

**Affiliations:** 1 Biomedical Informatics, State University of New York at Stony Brook, Stony Brook, NY, United States of America; 2 Radiology, State University of New York at Stony Brook, Stony Brook, NY, United States of America

**Keywords:** Serverless computing, Openhealth, Sparcs, Public health, Epidemiology data commons

## Abstract

In a previous report, we explored the serverless OpenHealth approach to the Web as a Global Compute space. That approach relies on the modern browser full stack, and, in particular, its configuration for application assembly by code injection. The opportunity, and need, to expand this approach has since increased markedly, reflecting a wider adoption of Open Data policies by Public Health Agencies. Here, we describe how the serverless scaling challenge can be achieved by the isomorphic mapping between the remote data layer API and a local (client-side, in-browser) operator. This solution is validated with an accompanying interactive web application (bit.ly/loadsparcs) capable of real-time traversal of New York’s 20 million patient records of the Statewide Planning and Research Cooperative System (SPARCS), and is compared with alternative approaches. The results obtained strengthen the argument that the FAIR reproducibility needed for Population Science applications in the age of P4 Medicine is particularly well served by the Web platform.

## Introduction

Three years ago we approached the feasibility of distributing interactive applications delivered entirely as in-browser constructs (Almeida et al., 2015). That software ecosystem was then described as “OpenHealth” with reference to the OpenData policy (Burwell et al., 2013). A multitude of BigData health-related resources has since become available, from the National Institutes of Health such as NCI’s Genome Data Commons (Wilson et al., 2017), to Population Health outcomes data collected by the health departments of a number of US states such as New York (NY. State of New York-Open Data Health-Health Data NY, 2018). Specifically, “OpenHealth applications” are assembled by code injection (JavaScript) and hosted with version control as github pages (gh-pages), which decouples the presentation layer from the logistics of data analysis and its governance (Almeida et al., 2015). That is, there are no servers to be maintained or applications to be downloaded and installed, which greatly extends the lifespan of the computational artifact. If the data sources are provided with regular updates, this lifespan is extended beyond the reported application to include the new data. The real-time deployment of OpenHealth applications can be confirmed by inspecting one of the original applications (bit.ly/pqiSuffolk) and verifying how the interactive analysis was updated with data made available years after the last update in the open source code.

The merits of the serverless approach have been well understood, and have been applied to biomedical data for a number of years, from genomics (Wilkinson & Almeida, 2014) to image analysis in pathology (Almeida et al., 2012). However, until recently it came with the suspicion that either the analytical challenge was computationally too intensive to be trackable as a client-side application, or that a dedicated server-side indexing resource would have to help carry the load. Interestingly, this perception that the performance of the “cloudification” (Bremer et al., 2016) of large data assets is challenged persists even when confronted with the favorable tabulation of execution times, as with did in that report at AMIA 2016. Instead, this architectural argument appears to be one that requires the development of “believe it when I see it” proof of concept applications that rely exclusively on the API of the data resource along the lines recently detailed for GDC, NCI Genomic Data Commons (Wilson et al., 2017). This argument, and the development of a validating application, were approached here by targeting Open Health Data resources of the Department of Health of New York State (NY. State of New York-Open Data Health-Health Data NY, 2018). In that data-intensive infrastructure, the core Data Commons argument that APIs with the ability to consume functionalized query languages are needed is addressed by SoQL (Socrata, 2018). On the one hand, this still falls short of the full Backend-as-a-Service (BaaS) model pursued by Data Commons (Grossman et al., 2016). On the other, because of the real-world shortcomings of public health data discussed later in this report, the Open Health Data offers the clearest practical assessment of the argument that the BaaS model is viable for any Data resource with a REST API able to consume query languages. This argument is currently the subject of a number of novel BaaS implementations, as detailed in the Discussion section.

Although the tool described in this report is being used at Stony Brook University Academic Medical Center to track signs as diverse as opioid overprescription or child obesity in Clinical Informatics bootcamps (Clinical Informatics Bootcamp, 2018), the purpose of this report is solely to describe the implementation methodology. Accordingly, only data in the public domain will be used and all code is provided with open source. Success in achieving this goal will be measured by the ability to deploy the interactive analytics application without requiring the direct management or hosting of servers. This approach to cloud computing where the web services are managed, and are assembled, as part of the cloud provision, is designated as “serverless” (Kanso & Youssef, 2017), in the sense that neither the application developer nor the user have to sustain them.

## Methods

### Architecture

**Figure 1:**
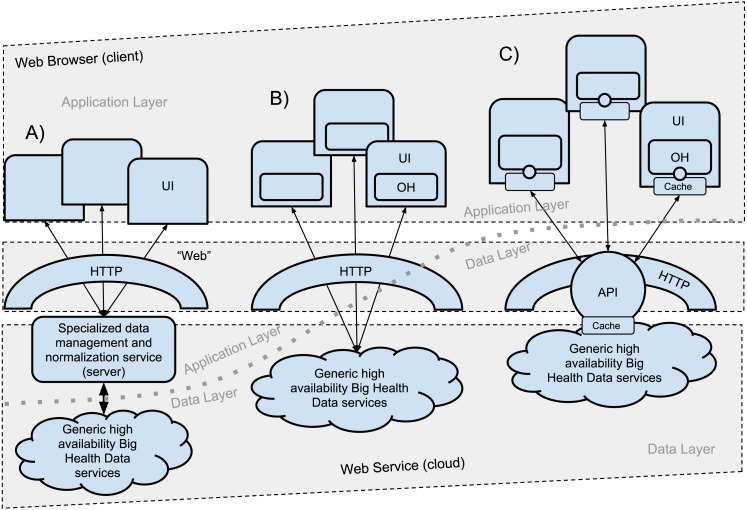
Evolving Web Computing Architectures. Evolution of the API economy from its pre-REST stage (A) to stateless transfer via HTTP (B), recently abstracted by constructs like GraphQL that combine an API language with a query engine (C). The prototype accompanying this report uses SoQL (see ‘Methods’) to illustrate the viability of the latter design, where the traversal of the Data Layer is abstracted as a stateless backend. The Cloud instantiation of this model approaches the description of BaaS (Backend-as-a-service).

The architecture design for this application starts with OpenHealth (Almeida et al., 2015), which is about in-browser constructs assembled on-the-fly by code injection, with the primary source of data served by remote HTTP-REST Application Programming Interfaces (API). That original implementation, recalled in Fig. 1B, followed the straightforward API Economy model (Brown, Fishenden & Thompson, 2014) of stateless integration by bringing together data from different sources via REST (Representational State Transfer) APIs. This is also the architecture where the ability to handle large amounts of heterogeneous data comes into question. Recalling from the introductory section, addressing this scaling challenge is best pursued with real-world health data sources, with real-world problems such as the lack of referential integrity that is often encountered in OpenData systems. Those practical challenges, the argument goes, would not be accurately assessed by applications targeting synthetic datasets or targetting heavily engineered BigData.

**Figure 2:**
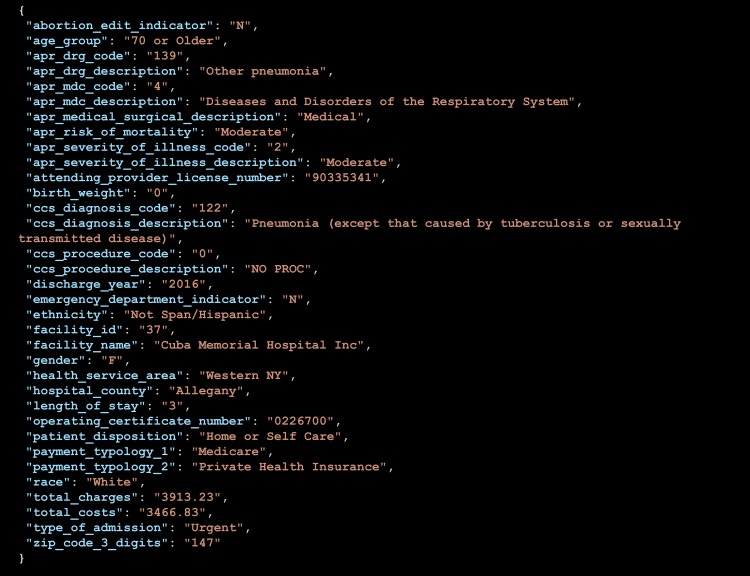
Snapshot of first of the 2,343,429 public records for 2016. See Table 1 for the full count. See also API section below for more information about why this exact public record can be programmatically retrieved from NY state Dept of Health: https://health.data.ny.gov/resource/gnzp-ekau.json?$limit=1.

### Data

The data used for this study is that of New York state Statewide Planning and Research Cooperative System (SPARCS) (NY. State of New York-Open Data Health-Health Data NY, 2018), made publicly available by the state’s Department of Health via SoQL APIs (Socrata, 2018). As detailed in the program’s web page at www.health.ny.gov/statistics/sparcs at the time of this writing, “*SPARCS is a comprehensive all-payer data reporting system established in 1979 as a result of cooperation between the healthcare industry and government. The system was initially created to collect information on discharges from hospitals. SPARCS currently collects patient level detail on patient characteristics, diagnoses and treatments, services, and charges for each hospital inpatient stay and outpatient (ambulatory surgery, emergency department, and outpatient services) visit; and each ambulatory surgery and outpatient services visit to a hospital extension clinic and diagnostic and treatment center licensed to provide ambulatory surgery services.*”

The public tier of the SPARCS dataset accessed by accompanying application documents 34 variables covering a range of parameters, from demographic and geographic to clinical, including payment information and identification of caregiver. Figure 2 provides a snapshot of the first entry of the over 2 million records for 2016. As the API section below details, ***this report and the accompanying application do not make any data available***: it simply distributes a in-browser computational artifact that engages the application programming interfaces of the Department of Health on behalf of the user (not the application developer). The flat file export of the SPARCS data alone (Table 1) is about 15 GB. Indexing its 34 fields to satisfy joint parameter constraints could have produced a far larger volume. The combination of size and combinatorial indexing are far in excess of what would have been possible to handle through client-side processing alone, the approach followed by the original OpenHealth model (Fig. 1B).

### API (application programming interface)

Table 1 lists all of the SoDA (Socrata, 2018) endpoints used by the accompanying application (see Availability). The document in reference details the API specification and the way in which Socrata provides interoperable Open Data infrastructure. For example, the record displayed in Fig. 2 can be obtained by dereferencing the address https://health.data.ny.gov/resource/gnzp-ekau.json?$limit=1.

### Availability of serverless application

The web application validating the serverless model (Fig. 1C) is available at bit.ly/loadsparcs (short link to https://mathbiol.github.io/#load%20sparcs). All code is available with open source and version control, both the base application at https://github.com/mathbiol/mathbiol.github.com and the sparcs module, at https://github.com/mathbiol/sparcs. All dependencies of this software are themselves also open source and, similarly to the accompanying application, only use JavaScript (EcmaScript) to ensure that no downloads or installations are needed. The latter is critical to explore the model and, specifically, how the code is able to travel to the computational scope of a user engaging a data source (Bell, Hey & Szalay, 2009). As discussed below, the unimpeded portability of the application signifies that it explores the scalability of controlled usage. Although the use of the application is what validates the results described in this report, a webcast video demo of traversing the SPARCS data is also available at mathbiol.github.io/sparcs/youtube. The inability to achieve real-time analytical interoperability at the SPARCS scale with the original OpenHealth architecture (Fig. 1B), and specifically what other constructs are emerging to support serverless (Fig. 1C) Data Commons, is further considered in the Discussion.

**Table 1:** Year, record count and public SPARCS data source traversed by the accompanying application. As the use of the application will make clear, these records come from all 58 counties of the state of New York.

**Year**	**# records**	**URL**
2009	2,665,414	https://health.data.ny.gov/resource/s8d9-z734
2010	2,622,133	https://health.data.ny.gov/resource/dpew-wqcg
2011	2,589,121	https://health.data.ny.gov/resource/n5y9-zanf
2012	2,544,543	https://health.data.ny.gov/resource/rv8x-4fm3
2013	2,428,500	https://health.data.ny.gov/resource/tdf6-7fpk
2014	2,367,283	https://health.data.ny.gov/resource/pzzw-8zdv
2015	2,346,760	https://health.data.ny.gov/resource/82xm-y6g8
2016	2,343,429	https://health.data.ny.gov/resource/gnzp-ekau
total:	19,907,183	https://www.health.ny.gov/statistics/sparcs/

## Results

**Figure 3:**
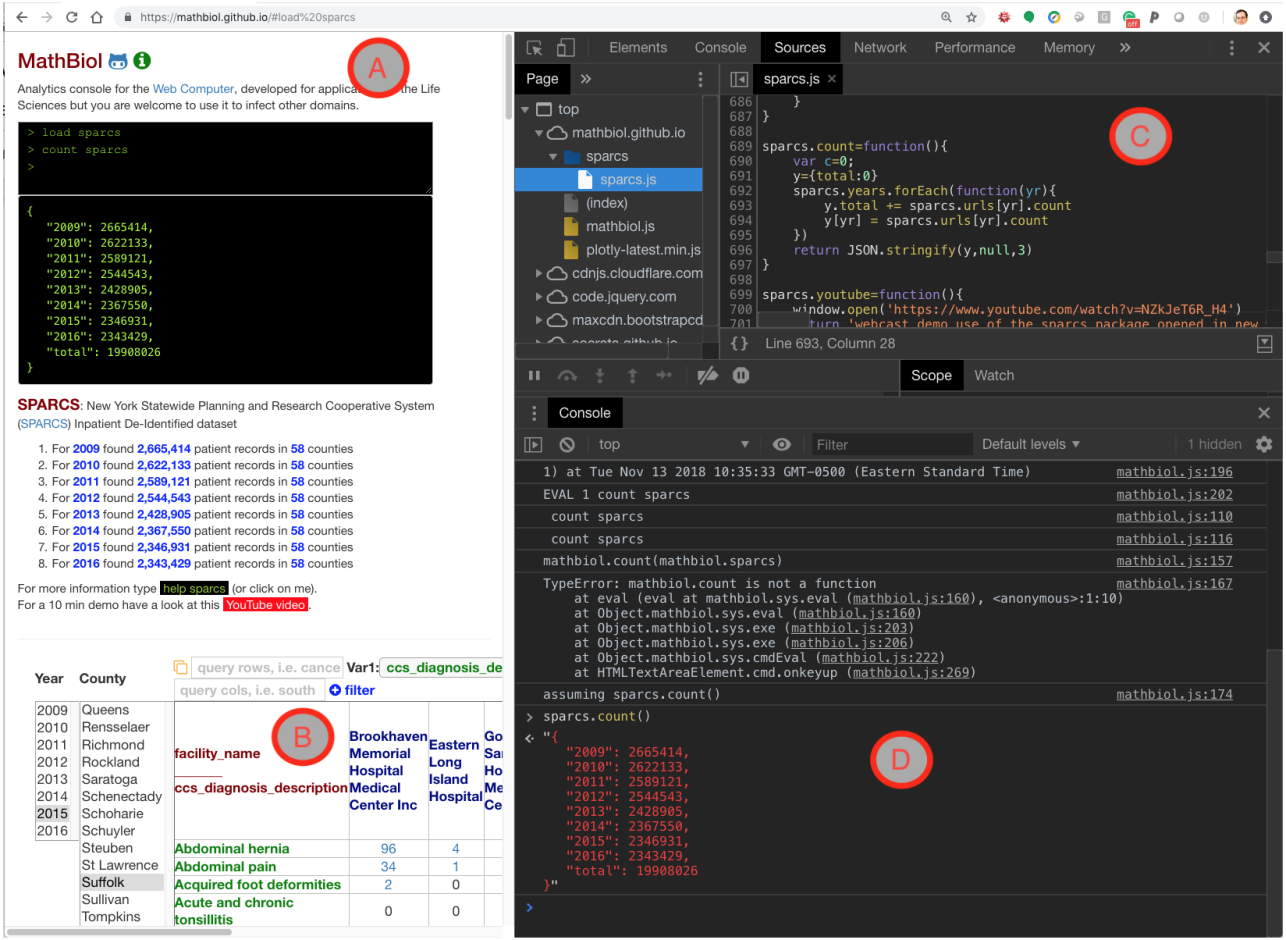
Snapshot of the SPARCS module loaded in Google Chrome Web browser with the developer tools open. Detail with developer tools open, on inspecting client-side methods operating a SoQL Socrata Query across all eight API endpoints (2009–2016) at NY’s Dept of Health. (A) shows the execution in the MathBiol console; (B) shows the same operator used to generate a list in HTML and the resulting table; (C) shows the code behind the count command, which migrated to the user’s browser from mathbiol.github.io/sparcs/sparcs.js (see Availability in ‘Methods’); Finally, (D) shows the same command being recognized after negotiating variations in the syntax (“TypeError”) used to call it. For clarity, the programmatic count call resulting from “assuming sparcs.count()” is also executed manually at the end of that negotiation. That is, the imprecise syntax of the command in the console (A) was caught (see error message in D) and an alternative syntax was found. This error catching approach allows for looser syntaxes in the user-interface (A), illustrating the opportunity to devise Domain Specific Languages (DSL).

At an architectural level, the SPARCS application was built on the foundations of the OpenHealth serverless model (Almeida et al., 2015). That architecture corresponds to a cached version of the Web 2.0 AJAX model described in Fig. 1B. As overviewed in the ‘Background’ section, the feasibility of that model is typically limited to applications that integrate moderate data volumes by operating the Data Layer API in a narrowly prescribed manner. This architecture was changed by creating a client-side object with attributes that map to the query language consumed by SoQL API, as explained in Fig. 1C. The key role of the isomorphic mapping of client-side methods to data-intensive server-side operations is illustrated in Fig. 3 for the count method used to generate the data in Table 1.

**Figure 4:**
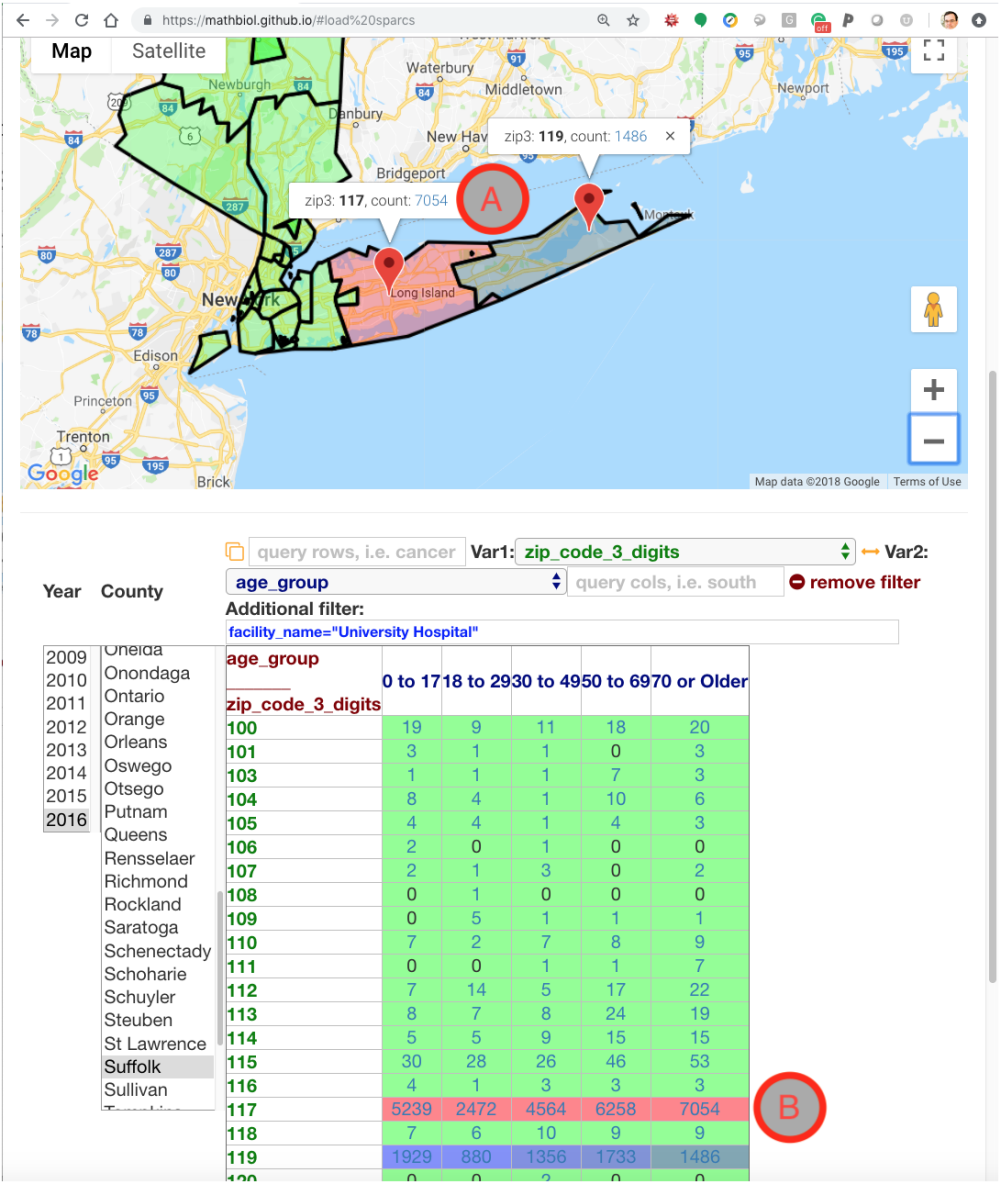
Snapshot of the SPARCS module in portrait mode in a mobile device, illustrating the ability to quickly resolve complex queries in moderately powered devices. Note how the graphic type responds to the data type: for example, the 3-digit zip code is matched by a geographic map display instead of a bar graph as in Fig. 3. The choice of variables can be compounded with additional constraints (additional filter), in order to, in this example, obtain the age groups and place of residence for patients seen at Stony Brook University Hospital. Each of the count numbers, underlined in blue, is a live link to the corresponding patient cohort. For example, clicking on “ 7054 ” either on the table (B) or in the map (A) will automatically retrieve the full data subset, with the values of all 33 parameters (Fig. 2) for each the 7,054 patients that satisfy the time, place and demographic constraints.

The snapshots in Figs. 3 and 4 illustrate the wide versatility of complex query constraints defined by the operation of the user interface, which is itself assembled in the user’s web browser without download or installation. That development versatility is the functionality that enables the BaaS model associated with the architecture described in Fig. 1C. However, the full measure of the BaaS model will be the operation of the APIs of remote data-intensive resources, as if they were local to the user’s own machine. That confirmation of scalability without loss of real-time interaction can only be verified by operating the application. See Availability in the ‘Methods’ section for the live web-based serverless application and demonstrative webcast video. The key role of the asynchronous NoSQL caching in the browser, IndexedDB, for web-based biomedical informatics has been noted by other researchers (Shi et al., 2015).

### Comparison with existing software tools

The development of mobile-first software to traverse open health data is still relatively new. As detailed in our original report on OpenHealth applications (Almeida et al., 2015), this reflects the early stage of development of consumer-facing software for outcomes-driven assessment of Health Care services. The key change is the public availability of large volumes of data-intensive resources that would have been considered too sensitive for publication just 2 years ago when the original OpenHealth tools were developed. Accordingly, two comparisons to existing tools are in order, speed and interactivity, while engaging the same SoQL API exposed by the Department of Health of the state of New York (health.data.ny.gov). The first comparison is straightforward: dereferencing a standard stateless application such as bit.ly/pqiSuffolk has a much longer assembly time, in the order to tens of seconds to a minute, than the approach presented here (Fig. 1C), bit.ly/loadsparcs, which takes less than 10 s and traverses a dataset over 100 times larger. The interactivity comparison is not as quantitatively straightforward because it requires the use of the analytical tools published with the data. That exercise can be approached by dereferencing, for example, health.data.ny.gov/Health/All-Payer-Hospital-Inpatient-Discharges-by-Facilit/srur-4jdu, and noting that the numerical results are not themselves linked to additional analysis where they are used as independent variables.

In summary, the proposed engagement of the data-intensive data-intensive SPARCS dataset has a clear advantage over approaches that do not use the cached BaaS model. That advantage is proposed here as a definite argument to approach data-intensive software Commons for research applications by using this model. That is, by mapping server-side to client-side abstractions as a generic backend that goes beyond the conventional stateless architecture of REST APIs. That conclusion, discussed at length in the next section, is particularly well aligned with recent developments in funding agencies promoting the use of interoperable cloud-hosted Research Commons infrastructure (Grossman, 2018b). Putting it plainly, the conventional “API economy”model (Figs. 1A–1B) simply doesn’t work as a client-side application at the SPARCS scale, regardless of the resources available to the machine used to run the web application. On the contrary, the new implementation (Fig. 1C) will work regardless of the machine, from high-end desktops to underpowered smartphones.

## Discussion

The objective of this coding exercise was to assess the viability of real-time traversal of real-world large health data resources. Lack of referential integrity caused by loose controlled vocabularies is amongst the most common and most challenging. Solving this problem *ex-post* (Hoekstra, 2010) in the presentation layer (in this case in the browser) is often considered an hopeless exercise because of a large number of records that would have to be fixed on-the-fly. Instead, mending referential integrity is typically addressed with ETL processes running in the data center. However, that objection may no longer be as relevant, because JavaScript engines have improved to the point of measuring themselves favorably with compilers in more conventional Data Science platforms. Case in point, close inspection of the SPARCS module reveals the use of MapReduce functional patterns, which may be executed in the machine’s Graphic Processing Units (GPU). It is noteworthy that modern browser includes native GPU APIs as part of its Document Object Model (DOM). It should also be noted that referential integrity in the SPARCS dataset is, as feared, broken by both loose variable naming conventions and value binning. To fix it, extensive corrections via Map operations are embedded in the *sparcs.getJSON* read operator, as detailed in the source code at https://github.com/mathbiol/sparcs/blob/master/sparcs.js#L34. In spite of the on-the-fly computation, there is no noticeable loss of interactivity of the SPARCS user-interface. Although not attempted here, this programmatic approach could be replaced by a more formal, declarative, approach to “sloppy data integration” (Almeida et al., 2006).

The Backend-as-a-Service (BaaS) model advanced by recent Data Commons infrastructure (Grossman et al., 2016) are recognized as the scalable route towards Precision Medicine (Jensen et al., 2017). Therefore, what combination of API language and query engine would best serve that goal in a FAIR manner (Wilkinson et al., 2016) is a critical design goal. In this study, SoQL (see Methods) was found to provide the necessary read-only interoperability. Naturally, the full BaaS model would require a more comprehensive approach to schema definition and data presentation. While this discussion is beyond the scope of the present report, it may be informative to note that data submission to NCI Genomic Data commons, at the time of this writing (as per GDC v1.13.0, Feb 18, 2018), requires the use of GraphQL as the interoperability model of choice for 3rd generation Data Commons infrastructure (Grossman, 2018a). In any case, new longitudinal Population Studies such as the NIH *All of Us* Research Program (National Institutes of Health, NIH), are bound to require a new approach to interactive analytics able to tackle the scale, diverse data models, and wide institutional distribution of associated cloud-based infrastructure for data-intensive science.

## Conclusion

The use of in-browser serverless applications (Web Apps calling data layer APIs directly) was tested with the real-world challenge of assembling web applications capable of traversing 20 million patient records of the public SPARCS dataset served by New York’s Department of Health. The portability and security of the web app model is a good match to the principles of FAIR Data Commons. The real-world test was that of interactive and open-ended constraint satisfaction on this large data space of well over half a billion individual measurements (34 × 19, 907, 183 = 676, 844, 222), convoluted by a significant lack of referential integrity. In spite of these obstacles, the isomorphic mapping of client-side operators to remote APIs supporting a full-fledged query language, combined with the native support for vectorized operators of the modern Web browser, was shown to achieve the performance levels required for real-time interactivity. It is therefore concluded that the emerging Data Commons frameworks are particularly well suited for ecosystems of Web applications. This BaaS behavior suggests a solution that overcomes the need for local, or even on-premise, implementations of Biomedical Informatics applications.

## Additional Information and Declarations

### Data Availability

The following information was supplied regarding data availability: GitHub: https://mathbiol.github.io/#load%20sparcs.

## References

[ref-1] Almeida JS, Chen C, Gorlitsky R, Stanislaus R, Aires-de-Sousa M, Eleutério P, Carriço J, Maretzek A, Bohn A, Chang A, Zhang F, Mitra R, Mills GB, Wang X, Deus HF (2006). Data integration gets ‘Sloppy’. Nature Biotechnology.

[ref-2] Almeida JS, Hajagos J, Crnosija I, Kurc T, Saltz M, Saltz J (2015). OpenHealth platform for interactive contextualization of population health open data. AMIA Annual Symposium Proceedings.

[ref-3] Almeida JS, Iriabho EE, Gorrepati VL, Wilkinson SR, Grüneberg A, Robbins DE, Hackney JR (2012). ImageJS: personalized, participated, pervasive, and reproducible image bioinformatics in the web browser. Jounal of Pathology Informatics.

[ref-4] Bell G, Hey T, Szalay A (2009). Computer science. Beyond the data deluge. Science.

[ref-5] Bremer E, Kurc T, Gao Y, Saltz J, Almeida JS (2016). Safe ‘cloudification’ of large images through picker APIs. AMIA Annual Symposium Proceedings.

[ref-6] Brown A, Fishenden J, Thompson M (2014). Digitizing Government: understanding and implementing new digital business models.

[ref-7] Burwell SM, VanRoekel S, Park T, Mancini DJ (2013). Memorandum for the Heads of Executive Departments and Agencies—managing Information as an asset. https://obamawhitehouse.archives.gov/sites/default/files/omb/memoranda/2013/m-13-13.pdf.

[ref-8] Clinical Informatics Bootcamp (2018). Stony Brook Dept of Biomedical Informatics. https://bmi.stonybrookmedicine.edu/bootcamp.

[ref-9] Grossman R (2018a). https://cdis.uchicago.edu/gen3/.

[ref-10] National Institutes of Health (NIH) (2018). All of us. https://allofus.nih.gov.

[ref-11] Grossman RL (2018b). Progress toward cancer data ecosystems. Cancer Journal.

[ref-12] Grossman RL, Heath A, Murphy M, Patterson M, Wells WA (2016). Case for data commons: toward data science as a service. Computing in Science & Engineering.

[ref-13] Hoekstra R (2010). The knowledge reengineering bottleneck. Semantic Web.

[ref-14] Jensen MA, Ferretti V, Grossman RL, Staudt LM (2017). The NCI genomic data commons as an engine for precision medicine. Blood.

[ref-15] Kanso A, Youssef A (2017). Serverless.

[ref-16] NY. State of New York-Open Data Health-Health Data NY (2018). New York State Department of Health—Health Data NY. https://health.data.ny.gov/.

[ref-17] Shi X, Peng J, Yu X, Zhang X, Li D, Liu B, Kong F, Yuan X (2015). PopGeV: a web-based large-scale population genome browser. Bioinformatics.

[ref-18] Socrata (2018). https://dev.socrata.com/docs/endpoints.html.

[ref-19] Wilkinson MD, Dumontier M, Aalbersberg IJ, Appleton G, Axton M, Baak A, Blomberg N, Boiten J-W, Da Silva Santos LB, Bourne PE, Bouwman J, Brookes AJ, Clark T, Crosas M, Dillo I, Dumon O, Edmunds S, Evelo CT, Finkers R, Gonzalez-Beltran A, Gray AJG, Groth P, Goble C, Grethe JS, Heringa J, ’t Hoen PAC, Hooft R, Kuhn T, Kok R, Kok J, Lusher SJ, Martone ME, Mons A, Packer AL, Persson B, Rocca-Serra P, Roos M, Van Schaik R, Sansone S-A, Schultes E, Sengstag T, Slater T, Strawn G, Swertz MA, Thompson M, Van der Lei J, Van Mulligen E, Velterop J, Waagmeester A, Wittenburg P, Wolstencroft K, Zhao J, Mons B (2016). The FAIR guiding principles for scientific data management and stewardship. Scientific Data.

[ref-20] Wilkinson SR, Almeida JS (2014). QMachine: commodity supercomputing in web browsers. BMC Bioinformatics.

[ref-21] Wilson S, Fitzsimons M, Ferguson M, Heath A, Jensen M, Miller J, Murphy MW, Porter J, Sahni H, Staudt L, Tang Y, Wang Z, Yu C, Zhang J, Ferretti V, Grossman RL (2017). Developing cancer informatics applications and tools using the NCI genomic data commons API. Cancer Research.

